# Vehicle configurations associated with anatomical-specific severe injuries resulting from traffic collisions

**DOI:** 10.1371/journal.pone.0223388

**Published:** 2019-10-07

**Authors:** Fumihito Ito, Yusuke Tsutsumi, Kazuaki Shinohara, Shunichi Fukuhara, Noriaki Kurita

**Affiliations:** 1 Department of Clinical Epidemiology, Fukushima Medical University, Fukushima City, Fukushima, Japan; 2 Center for Innovative Research for Communities and Clinical Excellence (CIRC2LE), Fukushima Medical University, Fukushima City, Fukushima, Japan; 3 Department of Sport Medicine, Fukushima Medical University, Fukushima City, Fukushima, Japan; 4 Department of Emergency Medicine, National Hospital Organization Mito Medical Center, Mito, Ibaraki, Japan; 5 Department of Healthcare Epidemiology, School of Public Health in the Graduate School of Medicine, Kyoto University, Kyoto, Japan; 6 Department of Emergency and Critical Care Medicine, Ohta Nishinouchi Hospital, Koriyama, Fukushima, Japan; 7 Department of Innovative Research & Education for Clinicians and Trainees (DiRECT), Fukushima Medical University Hospital, Fukushima City, Fukushima, Japan; Tongii University, CHINA

## Abstract

Vehicles can be classified by configuration as either bonnet-type or cab-over type according to engine location. Compared to bonnet-type, the front compartment of cab-over type vehicles is considerably shorter; thus, it may be less likely to absorb the energy generated in a collision, and in turn be unable to prevent deformation of the occupant space and protect occupants from injury. This study was a cohort study involving 943 occupants of mini-vehicles who were injured in frontal collision accidents between 2001 and 2015 and transferred to Ohta Nishinouchi Hospital. The vehicle configuration was divided into bonnet-type and cab-over type (i.e., truck-type and wagon-type). The tested outcomes were anatomical-specific severe injury of the pelvis and extremities, the head and neck, the abdomen, and the chest. To estimate adjusted odds ratios (AOR) for associations between vehicle configuration and anatomical-specific severe injury, we fitted generalized estimating equations for each outcome. Compared with bonnet-type vehicles, a greater risk of serious pelvis and extremities injury was found for both truck (AOR: 2.21; 95% Confidence Interval [95% CI]: 1.22–4.00) and wagon-type vehicles (AOR: 3.43; 95%CI 1.60–7.39). For serious head and neck injury, truck-type vehicles were associated with greater risk (AOR: 2.04; 95% CI: 1.10–3.79) than bonnet-type vehicles, whereas wagon-type vehicles were not. Compared with the occupants of bonnet-type vehicles, cab-over type vehicle occupants were more likely to have serious pelvis and extremities injury during frontal collisions. Additionally, truck-type vehicle occupants were more likely to have serious head and neck injury than bonnet-type vehicle occupants. These findings are expected to promote safer behaviors for vehicle occupants and the automobile industry.

## Introduction

**Fig 1:**
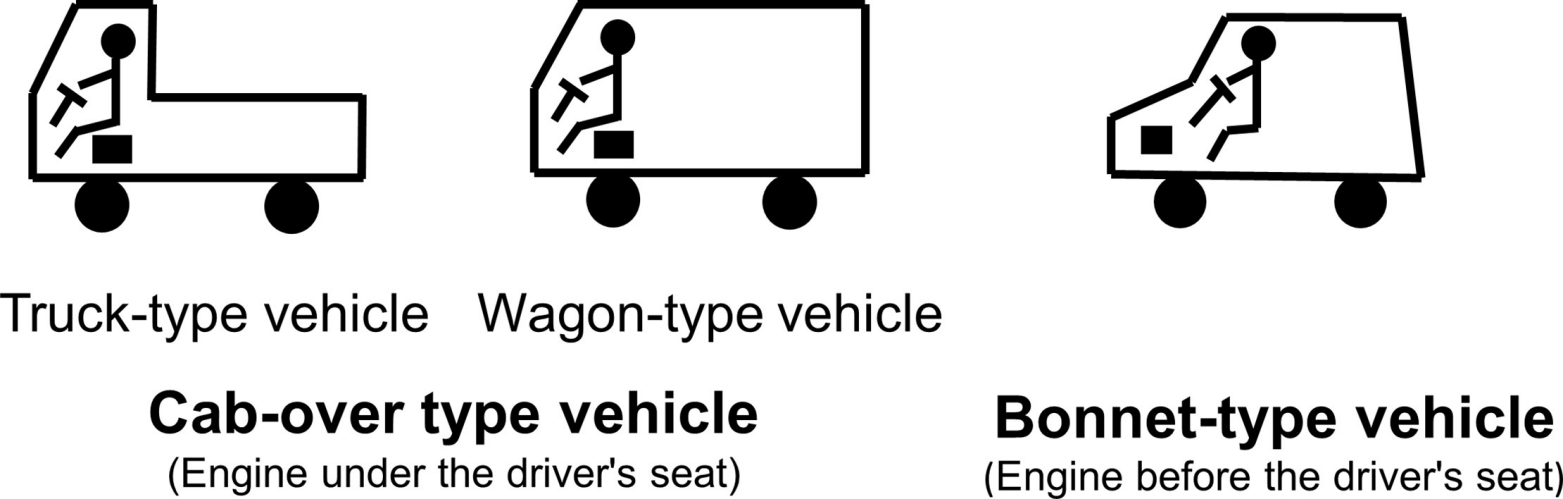
Vehicle configuration.

Road traffic accidents constitute a leading global cause of human fatalities and injury. Globally, the number of road traffic deaths is ~1.25 million per year. According to the Global Status Report on Road Safety 2015, published by the World Health Organization [1], road traffic injuries are the number one cause of death among 15–29 year-old individuals. One possible determinant of road traffic injury fatality is vehicle configuration, which can be classified as bonnet or cab-over type according to the engine location (Fig 1 and S1 Fig). Compared with that in bonnet-type vehicles, the front compartment of cab-over type vehicles are considerably shorter; thus, they may be less likely to absorb the energy generated in a collision, and in turn, less likely to prevent deformation of the occupant space and protect front occupants from injury. However, despite the widespread use of both vehicle types, evidence with regard to whether vehicle configuration is related to severe injury remains scarce.

Previous studies have reported that the cab-over type configuration is associated with a higher likelihood of severe anatomical injury [2,3]. A study from Korea reported that cab-over type vehicles are associated with more severe lower limb and abdominal injuries than bonnet-type vehicles [2]. However, this finding is confounded by the accident vehicles’ speed and the weights of the collided objects. Another study from Japan reported that the proportions of severe anatomical injuries differed among the vehicle occupants of cab-over and bonnet-type vehicles [3]. However, in that study, the differences in characteristics other than the vehicle configuration were not considered. Therefore, it is unclear whether anatomical-specific severe injuries are affected by vehicle configuration when appropriate safety equipment are used (e.g., airbags and seatbelts).

In this study, we examined whether differences in vehicle configuration contribute to anatomical-specific severe injuries using a cohort approach involving occupants injured in accidents in Fukushima, Japan (Reduction of Emergency and Death for Occupants of Collison Study (REDOCS)). The results of this study will allow car occupants to select safer vehicle types for purchase, and will assist the automobile industry in producing safer vehicle bodies.

## Materials and methods

### Study design and participants

This study was a cohort study involving vehicle occupants injured in frontal collision vehicle accidents and transferred to Ohta Nishinouchi Hospital, a tertiary emergency medical facility that covers the medical care zone for approximately 500,000 residents around Koriyama, Fukushima Prefecture. The accidents occurred between January 2001 and December 2015. We restricted our study to those injured in frontal collision accidents based on the assumption that vehicle configuration significantly influences the severity of the occupants’ injury only in frontal collisions. Further, only the occupants of mini-vehicles (Kei-car vehicles; defined as engine displacement ≤ 660 cc, vehicle length ≤ 3.4 m, width ≤ 1.48 m, and height ≤ 2.0 m according to the Japanese Road Transport Vehicle Act [4]) were considered. The mini-vehicle (Kei-car vehicle) is a vehicle class unique to Japan. This choice allowed us to minimize the influence of the vehicle size and weight on the occupants’ injury severity. Mini-vehicles are popular in Asia, especially in Japan [5]. We excluded occupants aged 16 years and under and occupants in rear seats.

### Exposure

The main exposure in this study was vehicle configuration divided into bonnet-type and cab-over type (Fig 1 and S1 Fig). Engines in bonnet-type vehicles are in front of the frontal seats, whereas engines in cab-over type vehicles are under the right frontal seat (driver's seat). The frontal compartment of a bonnet-type vehicle is longer than that of a cab-over type vehicle, owing to the different engine position. Cab-over type vehicles are subdivided into truck- and wagon-types. Truck-type vehicles have no rear seats and the back of the front seats is the loading platform. In contrast, wagon-type vehicles have rear seats.

### Outcomes

The outcomes of this study present data on anatomical-specific severe injuries of the pelvis and extremities, the head and neck, the abdomen, and the chest based on vehicle type. Injury severity was assessed according to the Abbreviated Injury Scale (AIS) coding system of 1990 (revision update 98) based on medical records and radiographic findings [6]. The AIS scores ranged from zero to six. All values were determined during hospital stay by a single well-trained trauma specialist and registered in medical records. We defined severe injuries as those with AIS = 3 or greater for each anatomical site.

### Covariates

Covariates were selected if a variable was potentially correlated with the vehicle configuration and outcomes. We collected demographics (age and sex), seat position (right front—the driver’s position in Japan—and left front), vehicle factors (seatbelt (seatbelt use or not), frontal airbag deployment (“equipped and deployment,” “equipped and non-deployment,” or “not equipped”), vehicle weight determined by car model (see S1 Appendix for details), self-reported vehicle speed before collision, and collided object (static object, vehicle with weight < 1t, vehicle with weight 1t ≤ to < 2t, or vehicle with weight ≥ 2t)). The category for vehicle weight in the collided object variable was determined based on the volume size of the vehicle (see S2 Appendix for details).

### Statistical analysis

The baseline characteristics (i.e., age, sex, vehicle factors, and collided object) were described by the mean and standard deviation (SD) for continuous variables and by the number and proportion (%) for categorical variables.

To estimate the odds ratios for associations between the vehicle configuration and the anatomical-specific injury severity, we separately fitted generalized estimating equations for each outcome, considering the clustering of occupants in the same accident using a robust variance estimator.

For each outcome, we conducted primary and sensitivity analyses. In the primary analysis, we entered all covariates into the fitted model and any missing values were imputed through multiple imputations using chained equations methods, assuming that the analyzed data were missing at random. The missing values were imputed using the existing AIS and other covariates. Five imputations were conducted. In the sensitivity analysis, we entered all covariates except for those with 30% or more missing data (vehicle speed before collision and vehicle weight) into the fitted model and conducted complete case analyses.

All statistical analyses were conducted using Stata^®^ version 14.0 (Stata Corp LP, College Station, TX). *P* values < 0.05 (two-tailed) were taken as indicators of statistical significance.

### Ethical consideration

In this study, anonymized data collected during routine practice were used. Informed consent was not mandatory, according to the ethical guidelines for epidemiological research in Japan. The study protocol was approved by the institutional review board of Ohta Nishinouchi Hospital (No. 11), Kyoto University Graduate School of Medicine (R0708), and Fukushima Medical University, School of Medicine (ippan-29004). The study was conducted in accordance with the Declaration of Helsinki and the ethical guidelines for epidemiological research in Japan.

The datasets generated during and/or analyzed during the study are available from the corresponding author on reasonable request.

## Results

**Fig 2:**
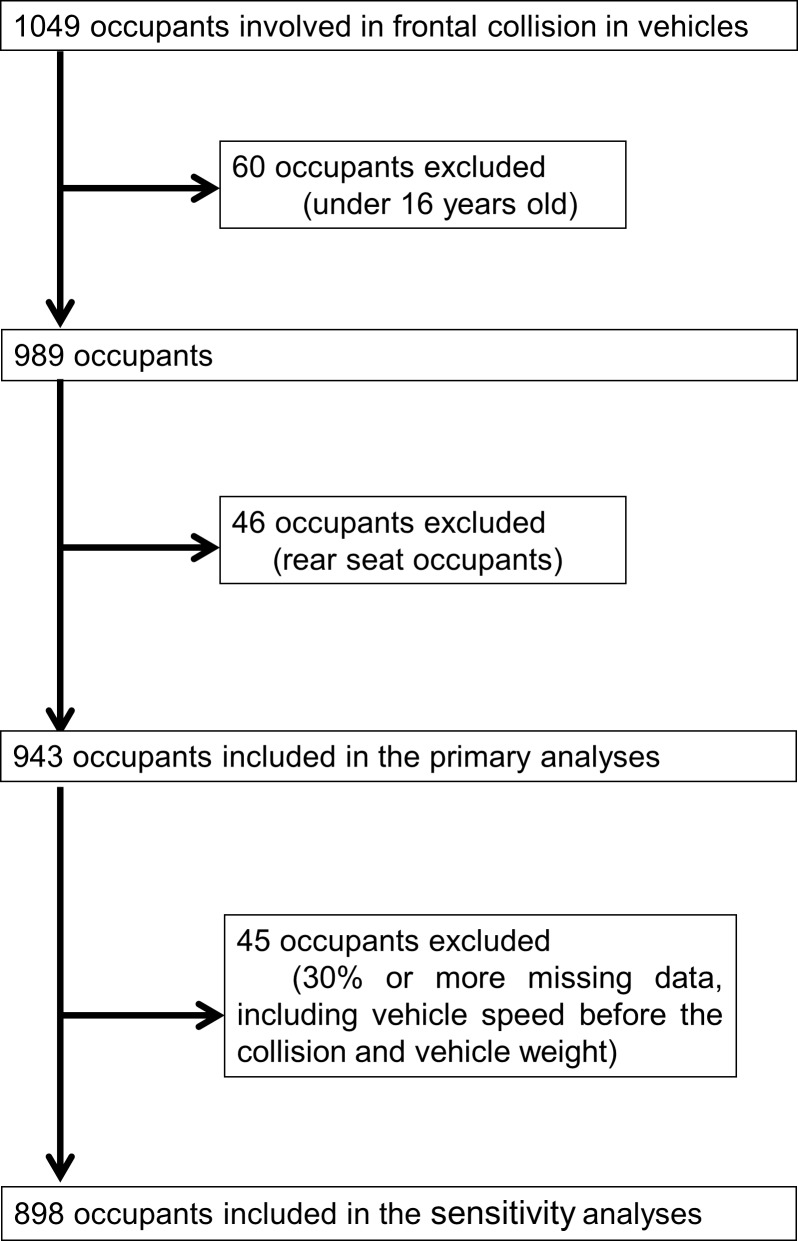
Flow chart of the study participant selection.

Of the 1049 occupants who were transferred because of frontal collision accidents over the 15 years, 60 were under 16 years old and 46 were rear occupants; all of these were excluded. Therefore, 943 occupants were included in our analyses (Fig 2).

Table 1 lists the characteristics of the vehicle occupants in the study. Of the occupants, 73% were in bonnet-type vehicles, and 27% were in cab-over type vehicles. Among the cab-over type occupants, 17% were truck-type occupants and 10% were wagon-type occupants. The mean age was 45.4 years.

**Table 1:** Characteristics of the vehicle occupants.

	Cab-over type (Truck)	Cab-over type (Wagon)	Bonnet-type	Total
	N = 159	N = 92	N = 692	N = 943
**Mean age (standard deviation)**	58.7 (17.3)	54.5 (16.7)	41.2 (19.3)	45.4 (20.1)
**Gender (as % Men)**	74%	75%	43%	51%
**Seat position (%)**				
Right frontal (driver's seat)	87%	86%	86%	86%
Left frontal	13%	14%	14%	14%
**Seat belts (%)**				
Belted	76%	79%	77%	77%
Missing (N = 28)	6	2	20	28
**Airbag (%)**				
Equipped and deployment	17%	42%	47%	42%
Equipped and non-deployment	3%	4%	15%	12%
Not equipped	80%	54%	38%	46%
Missing (N = 28)	7	3	18	28
**Characteristics of object collided with (%)**				
Static object	25%	33%	23%	25%
Vehicle weight < 1t	11%	13%	17%	16%
1t ≤ Vehicle weight < 2t	44%	33%	42%	44%
2t ≤ Vehicle weight	20%	17%	13%	15%
Missing (N = 3)	1	0	2	3
**Mean vehicle speed before collision [km/h] (standard deviation)**	40.7 (16.6)	46.1 (15.9)	45.9 (16.1)	(16.2)
Missing (N = 531)	97	56	378	531
**Mean vehicle weight [kg]** **(standard deviation)**	741 (30.5)	911 (70.7)	798 (77.7)	805 (83.1)
Missing (N = 266)	127	33	106	266
**Accident in which occupants simultaneously transferred to study facility (%)**				
1 occupant	93%	98%	95%	95%
2 occupants	7%	2%	5%	5%
**Occupants with anatomical-specific AIS ≥ 3 (%)**				
Pelvis and Extremity	19%	26%	9%	12%
Head and Neck	16%	13%	7%	9%
Abdomen	11%	9%	6%	7%
Chest	23%	21%	13%	15%

In total, 86% of the subjects were right frontal (driver’s seat) occupants, 77% were belted, and 46% were in vehicles not equipped with airbags. The mean vehicle speed before collision was 45.1 km/h, and the mean vehicle weight was 805 kg. The proportion of accidents in which one target occupant was transferred to the study facility was 95%. The proportions having anatomical-specific AIS = 3 or greater were 12%, 9%, 7%, and 15% for the pelvis and extremities, the head and neck, the abdomen, and the chest, respectively. Notably, the vehicle speed before collision and the vehicle weight had large proportions of missing values (56% and 28%, respectively).

The proportion of men in truck- and wagon-type vehicles was greater than that in bonnet-type vehicles. The bonnet-type vehicles had more frontal airbag equipment than the truck-type vehicles. The bonnet- and wagon-type vehicles had more frontal airbag deployments than truck-type vehicles.

The proportions of occupants in truck- and wagon-type vehicles with AIS = 3 or greater for the pelvis and extremities, the head and neck, and the chest, were higher than for the occupants of the bonnet-type vehicles.

**Fig 3:**
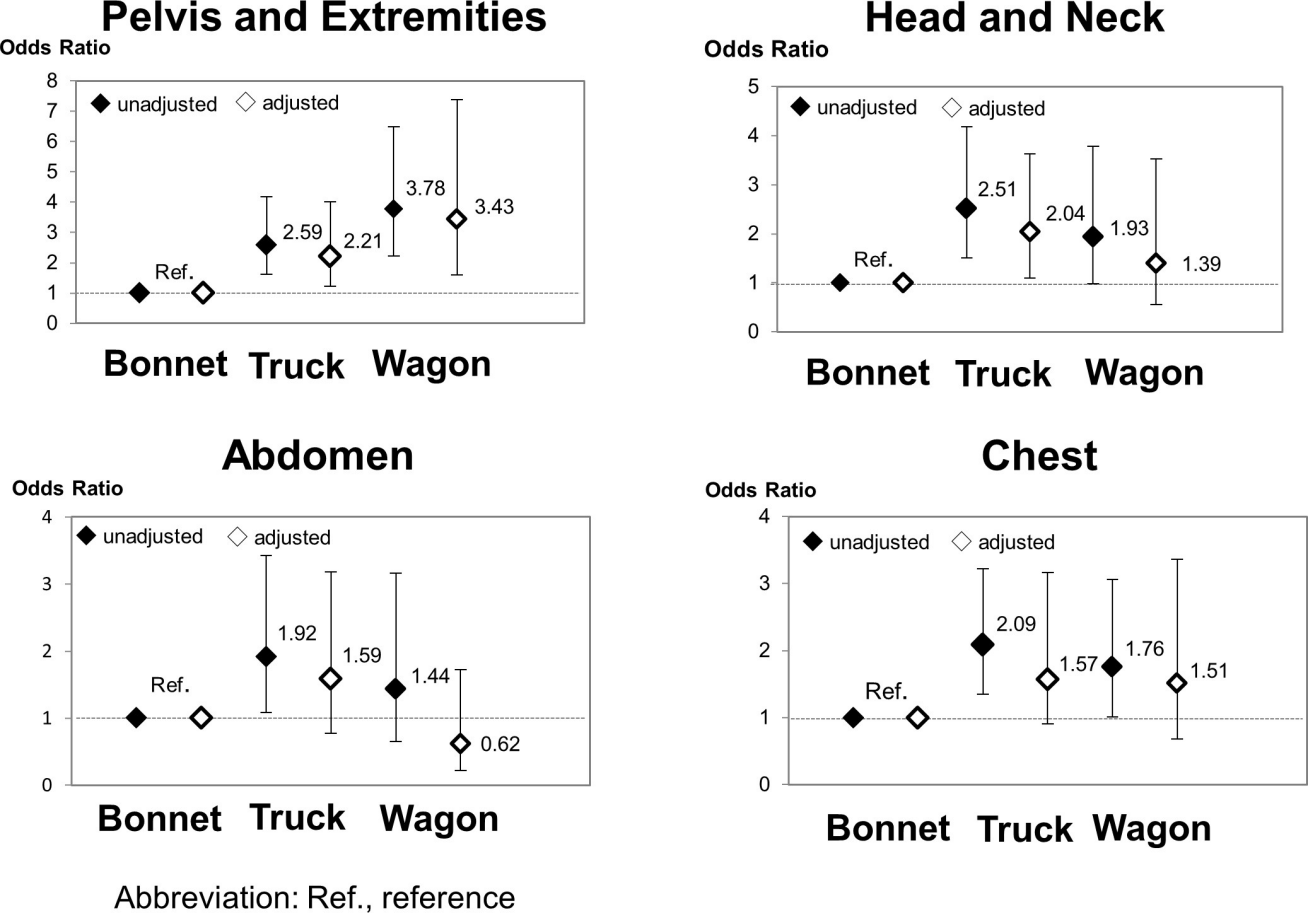
Association between the vehicle type anatomical-site specific AIS that is ≥ 3. Using multiply imputed data, generalized estimating equations were fitted to estimate the odds ratios for each anatomical specific Abbreviated Injury Scale (AIS), considering the clustering of occupants in the same vehicle with or without adjustment for age, sex, seat position, wearing a seatbelt, airbag deployment, weight of object collided with, vehicle speed before collision, and vehicle weight.

Table 2 and S1 Table and Fig 3 show the associations between the vehicle type and the anatomical-site specific AIS = 3 or greater, with adjustments for all covariates listed in Table 1 using multiply imputed data (N = 943). Compared with bonnet-type vehicles, a greater risk of serious pelvis and extremities injury was associated with both truck-type vehicles (adjusted odds ratio (AOR): 2.21; 95% Confidence Interval (95% CI): 1.22–4.00) and wagon-type (AOR: 3.43; 95% CI: 1.60–7.39). For serious head and neck injury, truck-type vehicles were associated with a greater risk (AOR: 2.04; 95% CI: 1.10–3.79) than bonnet-type vehicles, whereas wagon-type vehicles were not. For serious abdominal and chest injuries, cab-over type vehicles were not associated with greater risk than bonnet-type vehicles.

**Table 2:** Associations between vehicle type and anatomical-site specific severe injury (AIS 3 or greater).

	Bonnet-type	Cab-over type			
		Truck		Wagon	
Specific anatomical site		AOR	95% CI	AOR	95%CI
Pelvis and Extremity	Ref.	**2.21**	**1.22–4.00**	**3.43**	**1.60–7.39**
Head and Neck	Ref.	**2.04**	**1.10–3.79**	1.39	0.55–3.52
Abdomen	Ref.	1.59	0.77–3.26	0.62	0.22–1.72
Chest	Ref.	1.57	0.91–2.70	1.51	0.68–3.36

Abbreviation: Ref., reference; AOR, adjusted odds ratio; 95% CI, 95% confidence interval; AIS, Abbreviated Injury Scale (AIS).

Using multiply imputed data, generalized estimating equations were fitted to estimate the odds ratios for each anatomical-specific AIS, considering the clustering of occupants in the same vehicle, with adjustment for age, sex, wearing a seatbelt, airbag deployment, weight of object collided with, vehicle speed before collision, and vehicle weight.

The numbers in bold have a significant difference.

Table 3 shows the associations between the vehicle type and the anatomical-site specific AIS = 3 or greater with the adjustment of covariates listed in Table 1, except for the vehicle speed before collision and the vehicle weight (N = 898). The directions and the magnitudes of the associations are similar to those presented in Table 2.

**Table 3:** Associations between vehicle type and anatomical-site specific severe injury (AIS 3 or greater) without adjustment for vehicle speed before collision and vehicle weight.

	Bonnet-type	Cab-over type			
		Truck		Wagon	
Specific anatomical site		AOR	95% CI	AOR	95%CI
Pelvis and Extremity	Ref.	**2.06**	**1.15–3.68**	**3.79**	**2.03–7.03**
Head and Neck	Ref.	**1.95**	**1.05–3.65**	1.27	0.57–2.82
Abdomen	Ref.	1.89	0.96–3.74	0.93	0.38–2.28
Chest	Ref.	1.32	0.77–2.25	1.14	0.59–2.23

Abbreviation: Ref., reference; AOR, adjusted odds ratio; 95% CI, 95% confidence interval; AIS, Abbreviated Injury Scale (AIS).Using complete data with regard to all covariates except for vehicle speed before collision and vehicle weight, generalized estimating equations were fitted to estimate the odds ratios for each anatomical-specific AIS, considering the clustering of occupants in the same vehicle, with adjustment for age, sex, wearing a seatbelt, airbag deployment, and weight of object collided with.

The numbers in bold have a significant difference.

## Discussion

The results of this study show that occupants of cab-over vehicles (i.e., truck- and wagon-type vehicles) are more likely to have serious pelvis and extremities injury than bonnet-type vehicle occupants during frontal collisions. Additionally, truck-type vehicle occupants are more likely to have serious head and neck injury than bonnet-type vehicle occupants. These findings are expected to encourage vehicle occupants and the automobile industry to consider objective facts about safety during frontal collisions and to change their behaviors accordingly.

Our findings concur to some degree with the results of previous studies. A previous study from Japan reported that the proportion of severe injuries among cab-over type vehicle occupants was higher than that for bonnet-type vehicle occupants. These severe injuries were observed not only in the pelvis and extremities, but also in the head and neck, abdomen, and chest regions [3]. For example, the proportions of occupants having AIS ≥ 3 (i.e., severe injury defined similarly to our study) among cab-over type and bonnet-type occupants were 13.6% vs. 5.6%, 6.0% vs. 3.4%, and 11.9% vs. 8.5% for the pelvis and extremities, the abdomen, and the chest, respectively. However, their results were not adjusted for confounding factors such as seat belt use and airbag deployment. In our study, the proportions of occupants in vehicles not equipped with airbags were higher for the cab-over type than for the bonnet-type (Table 1). The lack of adjustment for airbag equipment in the previous study likely explains the differences in the results, with the proportions suffering from severe injuries of the abdomen and the chest being overestimated (Fig 3). In addition, the previous study failed to divide the cab-over type into truck- and wagon-type. However, by separating the truck-type from the wagon-type, we were able to show the magnitude of the association between truck-type (vs. bonnet-type) and severe injury of the head and neck differs from that of the association between wagon-type (vs. bonnet-type) and the severe injury.

Another study from Korea reported that the cab-over type was associated with more severe lower limb and abdominal injuries than the bonnet-type (AOR: 4.820 and 2.465, respectively) [2]. However, the associations in the study may have been confounded by the accident vehicle speed and the weights of the collided objects, and thus the magnitude of the associations may be overestimated. In our study, the proportions of collided objects in the heaviest weight category (2t ≤ weight) were higher among cab-over type vehicles than with bonnet-type vehicles (Table 1); therefore, the failure of adjustment for the collided objects in the previous study could have resulted in apparently stronger associations between the cab-over type and severe injury.

More importantly, the proportions of the cab-over vehicles deploying airbags were lower than those of the bonnet-type vehicles in the present study (Table 1). However, discrimination based on airbag status (i.e., whether “a vehicle is equipped with airbag but is not deployed” or “a vehicle is not equipped with airbag”) was not made in the previous study. As “Equipment and deployment of airbags” is a strong preventive factor for severe injury and death [7,8], this distinction and adjustment in the multivariable analysis is crucial. Thus, the stronger magnitude of the association between the cab-over type vehicles and severe injury in the Korean study than that in the present study may be explained by the potential dominance of vehicles not equipped with airbags in the cab-over group. We believe that our findings provide more robust results than those provided by the previous studies because we adjusted all important confounding variables. Finally, previous studies limited the cab-over type to the truck-type; the wagon-type was not examined in the Korean study. The inclusion of wagon-type vehicles is of greater importance, because wagon-type vehicles having occupants is more common than truck-type vehicles [9].

**Fig 4:**
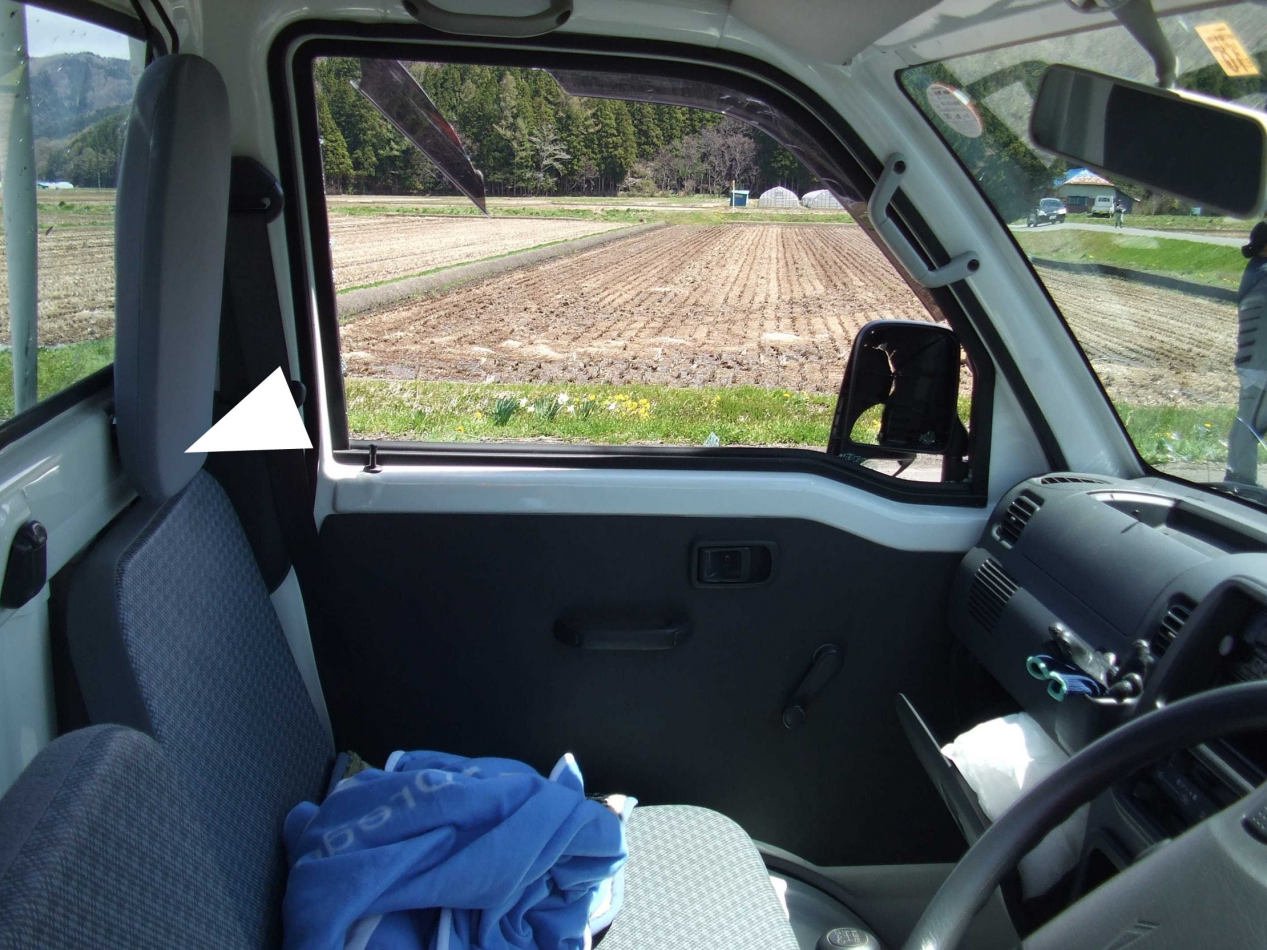
Seats of truck-type vehicles (the presence of a wall immediately behind the seats is shown by the arrowhead).

Some potential mechanistic reasons can explain our findings on severe injuries of the pelvis and extremities and the head and neck. First, the greater likelihood of having severe injuries of the pelvis and extremities among the cab-over type than among the bonnet-type could be attributed to the absence of the crash energy absorbing compartment in the cab-over type vehicles. The crash energy absorbing compartment of the bonnet lessens the energy generated in the event of a crash by denting; thereby, preventing deformation of the occupant space and injuries to the front occupants. The size of the crash energy absorbing compartment of cab-over-type vehicles is much smaller than that of bonnet-type vehicles, because the engine is located under the driver’s seat. In addition, some bonnet-type vehicles contain shock-absorbing materials in the bonnet space. Second, the greater likelihood of having severe head and neck injuries among the truck-type than those among the wagon- and bonnet-type vehicles may be attributable to the absence of a wall just behind the seat in the truck-type vehicles. The frontal seats of truck-type vehicles cannot move backward because of the wall (Fig 4); therefore, the heads of truck-type vehicle occupants are positioned further forward than those of wagon- and bonnet-type vehicle occupants. For this reason, truck-type vehicle occupants are more likely to move upwards with relation to the seatbelt rather than downwards at the time of a collision.

Our study has several strengths. First, our findings are derived from a large sample size, with analyses adjusted according to several important confounding factors (i.e., seatbelt, frontal airbag deployment, vehicle weight, self-reported vehicle speed before collision, and weights of the collided objects). Second, we obtained data not only for truck-type vehicles but also for wagon-type ones. Third, to mitigate the potential effects of the vehicle weight and vehicle size on severe injuries, our analyses focused on the occupants of mini-vehicles. Larger vehicles (e.g., passenger vehicles and SUVs) than mini-vehicles have greater variations in vehicle volume; thus, the effects of frontal shape may be greatly confounded by vehicle volume if we include the larger vehicles in the present analyses. In fact, mini-vehicles have become increasingly popular because they are convenient to drive and have economic benefits, including low purchase price, low tax, and good fuel economy. In 2017, mini-vehicles accounted for 39.1% of all registered vehicles in Japan [5]. The demand for mini-vehicles has spread worldwide. Both the importation of mini-vehicles, including used cars, from Japan and the local production of mini-vehicles are increasing among other countries in South Asia, East Asia, and Southeast Asia.

Our results have several implications for the public, medical personnel, and the automobile industry. First, our findings provide consumer safety information for the selection of vehicle type during vehicle purchase. Previous studies of vehicle preference among United States teenagers revealed that priority is given to vehicle price; thus, mini-vehicles, small vehicles, and/or old vehicles were preferred [10,11]. Many mini-vehicles adopt cab-over configurations to expand the internal space. The notification of the relatively higher risks associated with the cab-over vehicles than those with the bonnet-type vehicles could change consumer choices. Second, our findings may be applicable to pre-hospital settings for patient transfer. If an accident vehicle is a cab-over type, an ambulance crew can predict that the occupant may have severe internal injuries (e.g., pelvic injury) even if his/her external trauma does not seem to be severe; then, the crew can place a high priority on transferring the occupant to a level I trauma center. Third, our findings should encourage the automobile industry to invest in greater safety technology for cab-over vehicles. In Japan, all cab-over vehicle models must pass crash safety tests prior to sale. However, our results clearly show that cab-over vehicles are disadvantages relative to bonnet-type vehicles in frontal collisions, despite them passing the crash safety tests.

This study had several limitations. First, our findings are applicable only for mini-vehicles, they may not be generalizable to larger-sized vehicles. Further study is warranted to examine whether or not vehicle configuration is associated with severe injury for standard-sized vehicles. Second, our study setting was based on a tertiary emergency medical facility; this may give a skewed sample of patients in terms of injury severity. In general, patients with severe injuries are more likely to be transferred to tertiary emergency facilities; patients with slight injuries are less likely to be transported to such facility. Thus, proportions of severe injuries at a hospital will be higher than those seen at accident sites. However, we believe that associations between vehicle configuration and severe injuries are unlikely to be severely biased because the selection of patients transported to the hospital was determined by injury severity and irrespective of vehicle type at present. Third, the values of some covariates may be proxies. The velocity right before a collision is a self-reported measure. Regarding the vehicle weight, the values were based on standard equipment without consideration of the weights of occupants or cargo. Fourth, we were not able to collect the following variables in this study: driver's factors such as fatigue, fainting, and aggressive driving behavior; roadway factors such as light traffic, number of lanes, and hourly traffic volume; temporal factors such as traffic jam time, night, and weekend; and environmental factors such as rain, snow, wet road surface, snow road surface, ice road surface, and fog) [12–14]. While these variables are potential predictors of severe injuries, they do not necessarily meet the criteria for confounding in this study according to the classical epidemiologic framework. They do affect injury severity; however, they do so irrespective of the vehicle configuration in this study (i.e., these variables could not logically be used to determine the likelihood of a vehicle being cab-over type rather than bonnet-type). Further study is warranted to determine whether adjustment of these variables can alter the associations between vehicle configuration and injury severity examined in this study.

## Conclusions

This study found that occupants of cab-over mini-vehicle (i.e., truck- and wagon-type vehicles) occupants are more likely to have severe pelvis and extremities injury than bonnet-type mini-vehicle occupants during frontal collisions. In addition, truck-type mini-vehicle occupants are more likely to have severe head and neck injury than bonnet-type mini-vehicle occupants. Further study is required to determine if these findings extend to larger-sized vehicles.

## Supporting information

S1 Fig Vehicle configuration. (TIF)

S1 Appendix Calculation of vehicle weight. (DOCX)

S2 Appendix Classification of collided objects. (DOCX)

S1 Table Associations between vehicle type and anatomical-site specific AIS or more. (DOCX)
