# Supplementary figures and images for: Vehicle configurations associated with anatomical-specific severe injuries resulting from traffic collisions
Source: PLoS One. 2019 Oct 7;14(10):e0223388. doi: 10.1371/journal.pone.0223388 (PMC6779292; doi:10.1371/journal.pone.0223388)

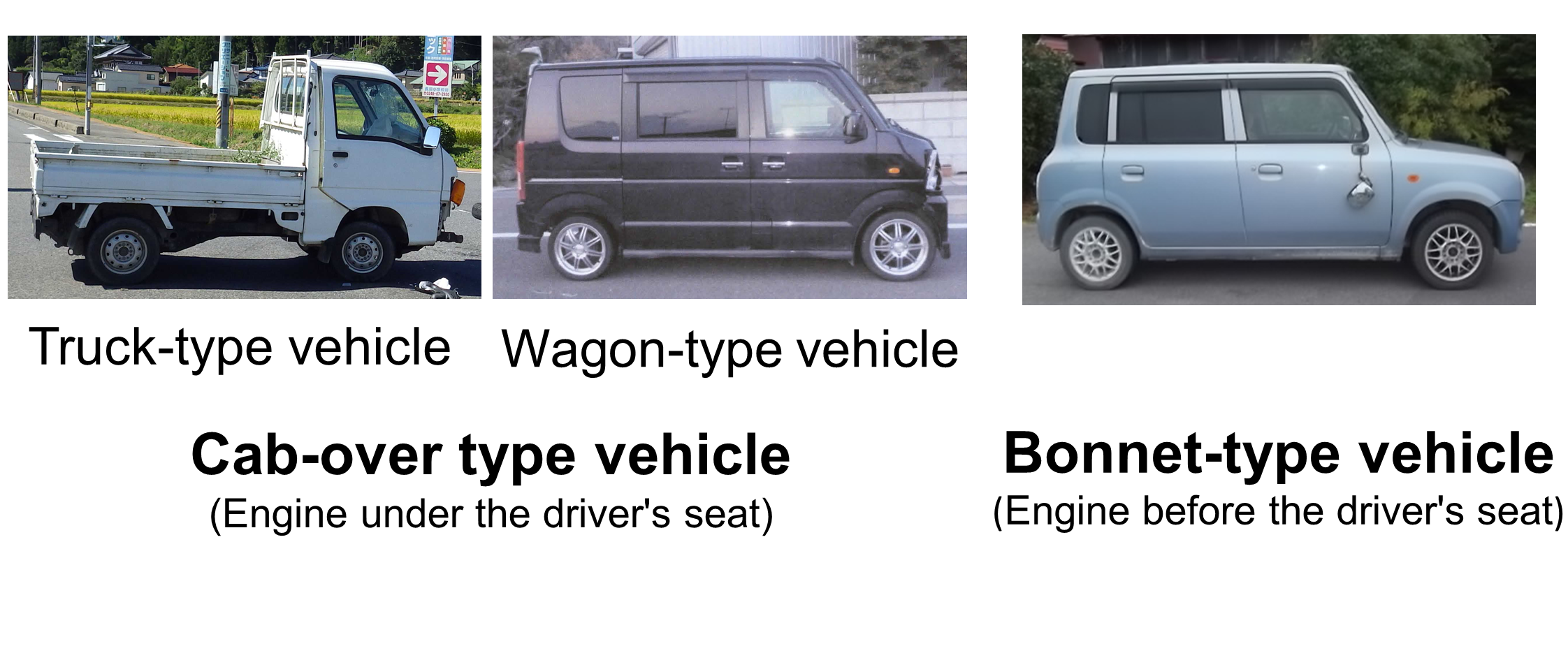

Supplement: S1 Fig — (TIF) [file pone.0223388.s001.tif]
