# Supplementary material for: Vehicle configurations associated with anatomical-specific severe injuries resulting from traffic collisions
Source: PLoS One. 2019 Oct 7;14(10):e0223388. doi: 10.1371/journal.pone.0223388 (PMC6779292; doi:10.1371/journal.pone.0223388)
Supplement: S1 Appendix — (DOCX) [file pone.0223388.s002.docx]

**S1 Appendix. Calculation of vehicle weight**

The vehicle weight used in this study was the average weight of the car model from the first year of production to the year of the accident.

For example, for a vehicle model that collided in 2007 and which was first marketed for sale in 2001, the average weight was calculated for that car model for the period between 2001 and 2007.

The weights of particular car models were extracted from the Cars Japan website [1]

**Reference**

1. Anonymous. Cars Japan: Cars Japan. 2018. Available from: <https://cars-japan.net/body/index.html>.
