# Supplementary material for: Vehicle configurations associated with anatomical-specific severe injuries resulting from traffic collisions
Source: PLoS One. 2019 Oct 7;14(10):e0223388. doi: 10.1371/journal.pone.0223388 (PMC6779292; doi:10.1371/journal.pone.0223388)
Supplement: S2 Appendix — (DOCX) [file pone.0223388.s003.docx]

**S2 Appendix. Classification of collided objects**

Four categories of collided objects were defined: vehicle with weights of ≥ 2t; vehicle with weights of 1t ≤ to <2t; vehicles with weights of <1t; and static objects.

Vehicles with weights of <1t corresponded to a mini vehicle (defined as engine displacement ≤ 660 cc, vehicle length ≤ 3.4 m, width ≤ 1.48 m, and height ≤ 2.0 m according to the Japanese Road Transport Vehicle Act [1] The vehicle weights of almost all the mini vehicles in our database were under 1t.

Vehicles with weights of ≥ 2t corresponded to trucks with a weight of 2 t or more, buses, and trailers. The weights of the trucks are registered in the database.

The vehicles with weights of 1t ≤ to < 2t corresponded to all vehicles classified as neither ‘Vehicle with weight <1t’ nor ‘Vehicle with weight ≥ 2t’ (e.g., the ‘futsu-jidosha’ and the ‘futsu-truck’).

The collided objects that did not move were classified as static objects.

Reference

1. Road Transport Vehicle Act [Available from: <http://elaws.e-gov.go.jp/search/elawsSearch/elaws_search/lsg0500/detail?lawId=326M50000800074#1869>.
