# Supplementary material for: Vehicle configurations associated with anatomical-specific severe injuries resulting from traffic collisions
Source: PLoS One. 2019 Oct 7;14(10):e0223388. doi: 10.1371/journal.pone.0223388 (PMC6779292; doi:10.1371/journal.pone.0223388)
Supplement: S1 Table — (DOCX) [file pone.0223388.s004.docx]

|  | Bonnet-type | Cab-over type | | | | | | |
| --- | --- | --- | --- | --- | --- | --- | --- | --- |
|  |  | Truck | |  | Wagon | | |  |
| Specific anatomical site |  | OR | 95%CI | | | OR | 95％CI | |
| Pelvis and Extremity | Ref. | **2.59** | **1.61** | **- 4.17** | **3.78** | | **2.21** | **- 6.49** |
| Head and Neck | Ref. | **2.51** | **1.51** | **- 4.18** | 1.93 | | 0.98 | - 3.78 |
| Abdomen | Ref. | **1.92** | **1.08** | **- 3.43** | 1.44 | | 0.65 | - 3.16 |
| Chest | Ref. | **2.09** | **1.35** | **- 3.22** | **1.76** | | **1.01** | **- 3.06** |

S1 Table. Association between vehicle type and anatomical-site specific AIS 3 or greater

Abbreviation: Ref., reference; AOR, adjusted odds ratio; 95% CI, 95% confidence intervals

No adjustment for covariates, while considering the clustering of occupants in the same vehicle. Generalized estimating equations are fitted to estimate the odds ratios for each anatomical specific AIS. The numbers in bold show a significant difference.
